# Caffeine Targets SIRT3 to Enhance SOD2 Activity in Mitochondria

**DOI:** 10.3389/fcell.2020.00822

**Published:** 2020-09-01

**Authors:** Huanhuan Xu, Chunxia Gan, Ziqi Gao, Yewei Huang, Simin Wu, Dongying Zhang, Xuanjun Wang, Jun Sheng

**Affiliations:** ^1^Key Laboratory of Pu-er Tea Science, Ministry of Education, Yunnan Agricultural University, Kunming, China; ^2^College of Science, Yunnan Agricultural University, Kunming, China; ^3^College of Food Science and Technology, Yunnan Agricultural University, Kunming, China; ^4^State Key Laboratory for Conservation and Utilization of Bio-Resources, Yunnan Agricultural University, Kunming, China

**Keywords:** caffeine, SIRT3, SOD2, antioxidant effects, UV radiation, skin photoprotection

## Abstract

Caffeine is chemically stable and not readily oxidized under normal physiological conditions but also has antioxidant effects, although the underlying molecular mechanism is not well understood. Superoxide dismutase (SOD) 2 is a manganese-containing enzyme located in mitochondria that protects cells against oxidative stress by scavenging reactive oxygen species (ROS). SOD2 activity is inhibited through acetylation under conditions of stress such as exposure to ultraviolet (UV) radiation. Sirtuin 3 (SIRT3) is the major mitochondrial nicotinamide adenine dinucleotide (NAD^+^)-dependent deacetylase, which deacetylates two critical lysine residues (lysine 68 and lysine 122) on SOD2 and promotes its antioxidative activity. In this study, we investigated whether the antioxidant effect of caffeine involves modulation of SOD2 by SIRT3 using *in vitro* and *in vivo* models. The results show that caffeine interacts with SIRT3 and promotes direct binding of SIRT3 with its substrate, thereby enhancing its enzymatic activity. Mechanistically, caffeine bound to SIRT3 with high affinity (*K*_D_ = 6.858 × 10^–7^ M); the binding affinity between SIRT3 and its substrate acetylated p53 was also 9.03 (without NAD^+^) or 6.87 (with NAD^+^) times higher in the presence of caffeine. Caffeine effectively protected skin cells from UV irradiation-induced oxidative stress. More importantly, caffeine enhanced SIRT3 activity and reduced SOD2 acetylation, thereby leading to increased SOD2 activity, which could be reversed by treatment with the SIRT3 inhibitor 3-(1H-1,2,3-triazol-4-yl) pyridine (3-TYP) *in vitro* and *in vivo*. Taken together, our results show that caffeine targets SIRT3 to enhance SOD2 activity and protect skin cells from UV irradiation-induced oxidative stress. Thus, caffeine, as a small-molecule SIRT3 activator, could be a potential agent to protect human skin against UV radiation.

## Introduction

Ultraviolet (UV)A and UVB radiation in sunlight negatively impacts the appearance of human skin. UVA is absorbed by chromophores in dermal cells to induce the generation of ROS that indirectly cause oxidative damage to DNA, leading to mutations and cancer (Farrar et al., 2018; Rybchyn et al., 2018). In contrast, UVB damages the membranes and proteins of cells on the surface layer of skin and superficial layer of the dermis, leading to sunburn erythema that can result in skin cancer in extreme cases (Khalil and Shebaby, 2017; Raad et al., 2017). ROS can activate the expression of MMPs that degrade the extracellular matrix including collagen and elastin fibers in the dermis, which can cause wrinkles, dandruff, and dullness (Burnstock et al., 2012; Ryu et al., 2019). Skin photoaging can be prevented by using sunscreen and other skincare products containing UV-absorbing compounds such as titanium dioxide, zinc oxide, salicylate, cinnamate, and anthranilate (Poh Agin, 2017).

Caffeine (1,3,7-trimethylxanthine) is an alkaloid found in many beverages and foods including tea, coffee, and cocoa that is an important natural secondary metabolite (Huang et al., 2014). The physical and chemical properties of caffeine make it highly stable, and evidence from *in vitro* and *in vivo* studies has shown that it can protect cells from oxidative stress and aging (Sutphin et al., 2012; Ma et al., 2015). For example, caffeine prevented acute ROS-induced necrosis in human skin fibroblasts and inhibited oxidative stress-induced aging by activating autophagy (Li et al., 2018). Additionally, caffeine was found to reduce oxidative stress by antioxidant mechanism (Badshah et al., 2019; Nilnumkhum et al., 2019).

Oxidative stress is caused by an excess of free radicals in cells and contributes to diseases such as aging and inflammation (Pesta and Roden, 2017). The redox homeostasis in mitochondria is tightly regulated by antioxidant enzymes such as SOD2 and glutathione peroxidase (Lu et al., 2017). It is well established that acetylation of SOD2 plays an important role in regulating its antioxidant activity (Liu et al., 2019). Yeast Sir2 protein and its homolog sirtuins in other prokaryotic and eukaryotic organisms belong to a class of protein deacetylases and ADP ribosylases with a highly conserved NAD^+^-binding core region (Schwer et al., 2002; Jin et al., 2009; Yi et al., 2019). In humans, there are at least seven Sir2-like proteins (SIRT1–7) with diverse functions including the regulation of chromatin structure and metabolism (Gonzalez Herrera et al., 2015; Fiskus et al., 2016). SIRT3 is the major mitochondrial NAD^+^-dependent deacetylase, and its activity was shown to be correlated with oxidative stress (Liu et al., 2015; van de Ven et al., 2017).

Under normal conditions, full-length SIRT3 (44 kDa) is present in the nucleus; upon exposure to external stimuli, it is targeted by MMPs and translocated to mitochondria as N-terminal SIRT3 (23 kDa) (Scher et al., 2007). Full-length SIRT3 has weak enzymatic activity, whereas the N terminus has strong NAD^+^-dependent deacetylase activity (McDonnell et al., 2015). The activity of the substrate-bound form of SIRT3 is altered by transfer of the acetyl group (Scher et al., 2007; Choudhary et al., 2009). SOD2 in the mitochondrial matrix is a deacetylated substrate of SIRT3, with acetylation at K68 and K122 playing crucial roles in its antioxidant activity (Singh et al., 2018; Zhu et al., 2019); additionally, an isothermal titration calorimetry (ITC) binding study showed that the acetylated peptide binds to SIRT3 before NAD^+^ (Jin et al., 2009). These findings suggest a relationship between deacetylation activity of SIRT3 and its role in redox homeostasis (Yang et al., 2016; Singh et al., 2018; Yi et al., 2019).

Based on the key role of SIRT3 in the regulation of redox homeostasis and the observation that caffeine activates A2AR/SIRT3/AMPK-mediated autophagy to protect skin from oxidative stress-induced aging (Li et al., 2018), we speculated that caffeine exerts antioxidant effects through SIRT3. In this study, we tested this hypothesis *in vitro* and *in vivo* using HaCaT immortalized human keratinocytes and a mouse model, respectively.

## Materials and Methods

### Chemicals and Reagents

Dimethyl sulfoxide was obtained from VWR Life Science (Solon, United States). DMEM was purchased from Gibco (Grand Island, NY, United States). Fetal bovine serum was sourced from Biological Industries (Beit HaEmek, Israel). Penicillin/streptomycin solution and a Modified Masson’s Trichrome Staining Kit (cat. no. G1345) were purchased from Solarbio (Beijing, China). Caffeine, DPPH (cat. no. 257621), and zinc diethyldithiocarbamate (cat. no. 329703) were obtained from Sigma-Aldrich (St. Louis, MO, United States). Phenol red-free medium (cat. no. 21063029), 5-(6)-chloromethyl-2′,7′-dichlorodihydrofluorescein diacetate, acetyl ester (CM-H_2_DCFDA; cat. no. C6827), and MitoSOX Red mitochondrial superoxide indicator (cat. no. M36008) were purchased from Thermo Fisher Scientific (Waltham, MA, United States). 3-TYP (cat. no. S8628) was purchased from Selleck Chemicals (Houston, TX, United States). An Annexin V-FITC/PI kit was purchased from Beijing 4A Biotech Co., Ltd. (Beijing, China). Antibodies against SIRT3 (cat. no. 5490), cleaved caspase3 (cat. no. 9664), Bcl-2 (cat. no. 15071), and Bax (cat. no. 2772) were obtained from Cell Signaling Technology (Beverly, MA, United States). Antibodies against SOD2 (cat. no. ab68155), SOD2-K68-Ac (cat. no. ab137037), SOD2-K122-Ac (cat. no. ab214675), cleaved poly (ADP-ribose) polymerase (PARP)1 (cat. no. ab32064), and MMP3 (cat. no. ab52915) were purchased from Abcam (Cambridge, United Kingdom). The antibody against β-tubulin (cat. no. 100109-MM05T) was purchased from Sino Biological Inc. (Beijing, China). SIRT3 Direct Fluorescent Screening Assay Kit (cat. no. KA1369), Superoxide Dismutase (SOD) Activity Assay Kit (cat. no. S311), and H&E Staining Kit were purchased from Abnova (Taiwan, China), Dojindo (Shanghai, China), and Beyotime Biotechnology (Shanghai, China), respectively.

### DPPH Assay

The *in vitro* antioxidant activity of caffeine was measured with the DPPH assay as previously described (Kumar and Mandal, 2017; Hermankova et al., 2019). Briefly, a 2-mL aliquot of each sample solution was added to 2 mL of DPPH solution in a quartz cuvette, yielding final solutions with different concentrations of caffeine (32–4,096 μM) and 0.2 mM DPPH. The absorbance at 517 nm was measured in triplicate for each solution with a UV-visible spectrophotometer (Multiskan MK3; Thermo Fisher Scientific). Radical scavenging activity was determined by comparing the absorbance of DPPH after reduction by an antioxidant (yellow color) with that of the blank (deep purple color).

### *In vitro* SIRT3-Mediated SOD2 Deacetylation Assay

To perform *in vitro* SIRT3-mediated SOD2 deacetylation assay, the acetylated SOD2 was extracted from UV-irradiated HaCaT cells. Briefly, HaCaT cells were irradiated with UVB lamp for 50 min, then lysed, and finally immunoprecipitated with SOD2 antibody. The purified acetylated SOD2 was incubated with SIRT3 in the absence or presence of caffeine for 5 min at 37°C in 50 mM Tris-HCl (pH 7.5), 300 μM NAD^+^ buffer. Subsequently, the reaction mixture was subjected to western blotting using primary antibodies against SOD2-K68-Ac, SOD2-K122-Ac, and SOD2.

### Biolayer Interferometry (BLI) Assay

The binding affinity of SIRT3 with caffeine was determined using Super Streptavidin (SSA) biosensors in an Octet Red 96 instrument (ForteBio Inc., Menlo Park, CA, United States). Briefly, the biotinylated SIRT3 protein was immobilized onto the surface of SSA biosensors. Increasing concentrations of caffeine were allowed to interact with the immobilized SIRT3 at 25°C in phosphate-buffered saline (pH 7.4). The final volume of all solutions was 200 μL. Assays were performed in black solid 96-well flat bottom plates on a shaker set at 1,000 r/min. The association and dissociation of caffeine with SIRT3 was measured for 300 s each. Kinetic parameters and affinities were calculated from a non-linear global fit of the data between caffeine and SIRT3 using Octet Data Analysis software version 7.0 (Fortebio).

### Surface Plasmon Resonance (SPR) Studies

The SPR experiments were carried out using a Biacore S200 instrument (Biacore, GE Healthcare) at 25°C. SIRT3 (30 μg/mL in 10 mM sodium acetate, pH 4.0) was immobilized on the flow cell of the Series S CM5 Sensor Chip using an amine coupling kit (GE Healthcare). The analytes, including caffeine (6.24–0.39 μM, double dilution), NAD^+^ (6.24–0.39 μM, double dilution), and acetylated p53 (200–12.5 μM, double dilution), were injected and passed over the immobilized SIRT3 sensor surface. In addition, acetylated p53 (25–1.56 μM or 6144–24 nM, double dilution) was also injected and passed over the chip surface in the presence of caffeine (1 μM), NAD^+^ (1 μM), and caffeine plus NAD^+^, respectively. The flow rate was 30 μL/min, the binding time was 90 s, and the dissociation time was 90 s. Kinetics and affinity analyses were performed using Biacore S200 Evaluation Software (version 1.1, GE Healthcare).

### Cell Culture

Human HaCaT cells were obtained from Conservation Genetics CAS Kunming Cell Bank (Kunming, China) and cultured in DMEM containing 10% fetal bovine serum and 1% penicillin/streptomycin solution in a humidified incubator at 37°C and 5% CO_2_.

### Animal Care

Female C57BL/6 mice (8 weeks old) were purchased from Cawens Lab Animal Co. (Changzhou, China). The animals were housed in a room with controlled temperature (23°C ± 2°C), humidity (50% ± 5%), and illumination (12:12-h light/dark cycle), and were allowed to adapt to the facility for 2 weeks before experiments. All animal experiments were approved by the Institutional Animal Care and Use Committee of the Yunnan Agricultural University (Approval Number: YNAU2019LLWYH003), and were performed in accordance with the National Institutes of Health’s Guide for the Care and Use of Laboratory Animals (NIH publication No.80-23, revised in 1978).

### UV Irradiation

A total of 42 mice were divided into the following seven groups: control, UV alone, caffeine (1.29 mM) + UV, caffeine (5.15 mM) + UV, caffeine (1.29 mM) + 3-TYP (13.68 nM) + UV, caffeine (5.15 mM) + 3-TYP (13.68 nM) + UV, and 3-TYP (13.68 nM) + UV. For animal studies, skins were irradiated using Philips UVA and UVB lamps (Philips, Amsterdam, Netherlands). The doses of UVA and UVB were, respectively, equivalent to 15.975 and 5.325 J/cm^2^ for the first day and decreased to 12.975 and 4.325 J/cm^2^ in the next 6 days. Total irradiated doses of UVA and UVB were approximately 125 J/cm^2^. Skins were smeared with different doses of caffeine or 3-TYP twice daily 1 h at 30 min intervals before UV exposure. We used a self-made solution consisting of propylene glycol and absolute ethanol at a volume ratio of = 7 to 3 as a vehicle for drug dissolution and dosing. After the last UV exposure, dorsal skin tissues were collected for H&E and Masson’s trichrome staining, detection of SIRT3 and SOD2 activities, and western blotting. For cultured HaCaT cells, the UVB irradiation dose was 0.53 J/cm^2^.

### Cell Viability Assay

The effects of caffeine on cell growth was evaluated with the MTT assay. Briefly, HaCaT cells were seeded at 1.5 × 10^4^ cells/well in a 96-well plate and cultured overnight, then treated with different concentrations of caffeine (128–512 μM) for 24 h. After removing the supernatant, DMSO was added to the wells and the optical density at 492 nm was measured with a microplate reader (FlexStation 3; Molecular Devices, Sunnyvale, CA, United States). Cell viability was normalized to that of control cells.

### Crystal Violet Staining

Viable HaCaT cells (1 × 10^6^ cells/plate) were seeded in 60-mm plates and incubated overnight. The following day, the cells were pretreated with caffeine (256 or 512 μM) for 1 h in the serum-free medium and subsequently irradiated with UVB lamp for 5 min. After 24 h, the cells were fixed with 4% paraformaldehyde for 20 min and stained with crystal violet for 10 min before they were photographed.

### SIRT3 Activity Assay

The effect of caffeine on the deacetylase activity of SIRT3 was evaluated with the SIRT3 Direct Fluorescent Screening Assay Kit according to the manufacturer’s instructions. Briefly, after initiating the reactions by adding 15 μL of substrate solution, fluorescence was measured with the Multiskan MK3 spectrophotometer at excitation and emission wavelengths of 355 and 460 nm, respectively.

For adherent HaCaT cells, 8 × 10^5^ viable cells were cultured in 60-mm plates until they reached 50% confluence. The cells were pretreated with caffeine (256 or 512 μM) for 1 h and subsequently irradiated with UVB lamp for 5 min, then lysed by adding KDalert lysis buffer from the kit, and finally immunoprecipitated with SIRT3 antibody. The remaining steps were performed according to the manufacturer’s protocol.

For animal skin tissues, the sample was weighed and a 16× volume of KDalert lysis buffer was added for 5 min, after which the sample allowed to stand on ice for 5 min. This process was repeated four times, and the sample was centrifuged at 16,000 × *g* and 4°C (Hitachi, Tokyo, Japan), yielding a clarified protein solution; the protein extracts were immunoprecipitated with SIRT3 antibody and then used for SIRT3 activity assay. At least three replicates of each sample were prepared.

### SOD2 Activity Assay

SOD2 activity in HaCaT cells or animal skin tissues was detected with the SOD Activity Colorimetric Assay Kit according to the manufacturer’s instructions. Briefly, zinc diethyldithiocarbamate (final concentration: 1 mM) was added to the sample to inhibit the activity of Cu/Zn-SOD and extracellular SOD (Heikkila et al., 1976; Lu et al., 2017). Different samples were added to the experimental and blank wells, and a constant final volume was maintained by adjusting the buffer volume. The absorbance at 450 nm was measured.

### Detection of Cellular and Mitochondrial ROS

Intracellular ROS level was detected with CM-H_2_DCFDA (Shah et al., 2013; Baek et al., 2017). HaCaT cells were seeded in a 12-well plate at a density of 1 × 10^5^ cells/well. When they reached 50% confluence, the cells were pretreated with caffeine (256 or 512 μM) for 1 h and subsequently irradiated with UVB lamp for 5 min. After 24 h, the medium was replaced with 5 μM dye (1 mL) in phenol red-free medium followed by incubation for 15 min, and images were acquired at200× magnification under a fluorescence microscope (Leica Microsystems, Wetzlar, Germany). Intracellular ROS appeared as green fluorescence under the microscope. A similar procedure was used to detect mitochondrial ROS except that MitoSOX dye was used, which was visible as red fluorescence. The images were analyzed using ImageJ software (National Institutes of Health, Bethesda, MD, United States).

### Cell Apoptosis Assay

Cell apoptosis was determined using an Annexin V-FITC/PI kit according to the manufacturer’s instructions. HaCaT cells were pretreated with caffeine (256 or 512 μM) for 1 h and then irradiated with UVB lamp for 5 min. After 24 h, the cells were collected and incubated in 200 μL of 1× binding buffer and 20 μL of Annexin V-FITC, followed by staining with 20 μL of PI for 15 min at room temperature in the dark. Subsequently, the cells were subjected to flow cytometry (BD FACSCalibur, PA, United States) analysis within 1 h.

### Western Blotting

Cells were lysed in radioimmunoprecipitation assay (RIPA) lysis buffer containing 1% phenylmethylsulfonyl fluoride (Solarbio) for 30 min on ice and the lysate was collected by centrifugation (15,000 × *g* for 10 min at 4°C). Protein concentration was quantified using an Enhanced BCA Protein Assay Kit (Beyotime Biotechnology). Equal amounts of proteins were loaded and separated by 12% sodium dodecyl sulfate polyacrylamide gel electrophoresis (SDS-PAGE), then transferred to polyvinylidene difluoride membranes (Millipore, Billerica, MA, United States) that were incubated with the indicated primary antibodies followed by horseradish peroxidase-conjugated anti-rabbit or -mouse secondary antibody. The bands of interest were detected using an Ultra-sensitive Enhanced Chemiluminescent Substrate Kit (Beijing 4A Biotech Co., Ltd). β-tubulin was used as the loading control. Relative protein expression was quantified using AlphaView software (Cell Biosciences, Santa Clara, CA, United States). As for animal skin tissues, the protein lysates were performed for western blotting.

### Pharmacological Inhibition of SIRT3 Activity by 3-TYP

3-TYP is an inhibitor of SIRT3 activity with a half-maximal inhibitory concentration of 16 nM (Ye et al., 2019). HaCaT cells were pretreated with 3-TYP in the absence or presence of caffeine for 1 h and then irradiated with UVB lamp for 5 min. After 1 h, the cells were used for SIRT3 and SOD2 activities assay, mitochondrial ROS detection, and western blotting.

### Histological Analysis

Mouse skin tissues were isolated and fixed in formalin for over 24 h, then dehydrated, embedded in paraffin, and cut into sections at a thickness of 5 μm. Changes in the thickness of the stratum corneum were evaluated by H&E staining. The nucleus appeared as dark blue; the cytoplasm, muscle, and connective tissue were red. Changes in collagen content of the dermis were assessed by Masson’s trichrome staining, yielding a blue-violet nucleus; red cytoplasm, glial fibers, keratin, and muscle; and blue collagen fibers. Images were captured at 100× magnification with a Leica microscope and Leica CellSens Dimension software.

### TUNEL Assay

The level of skin tissue apoptosis *in vivo* was detected using a YF^®^488 TUNEL assay apoptosis detection kit (US Everbright^®^Inc., Suzhou, China) according to the manufacturer’s protocol. The fluorescence microscopy images were taken at a 100× magnification.

### Statistical Analysis

Data were analyzed and plotted using Prism 5.0 software (GraphPad, La Jolla, CA, United States) and are presented as the mean ± SEM of at least three independent experiments. Statistically significant differences between groups were evaluated with the Student’s *t*-test. A *P*-value less than 0.05 was considered statistically significant.

## Results

### Caffeine Does Not Have Antioxidant Activity *in vitro* but Promotes SIRT3 Activity

**FIGURE 1:**
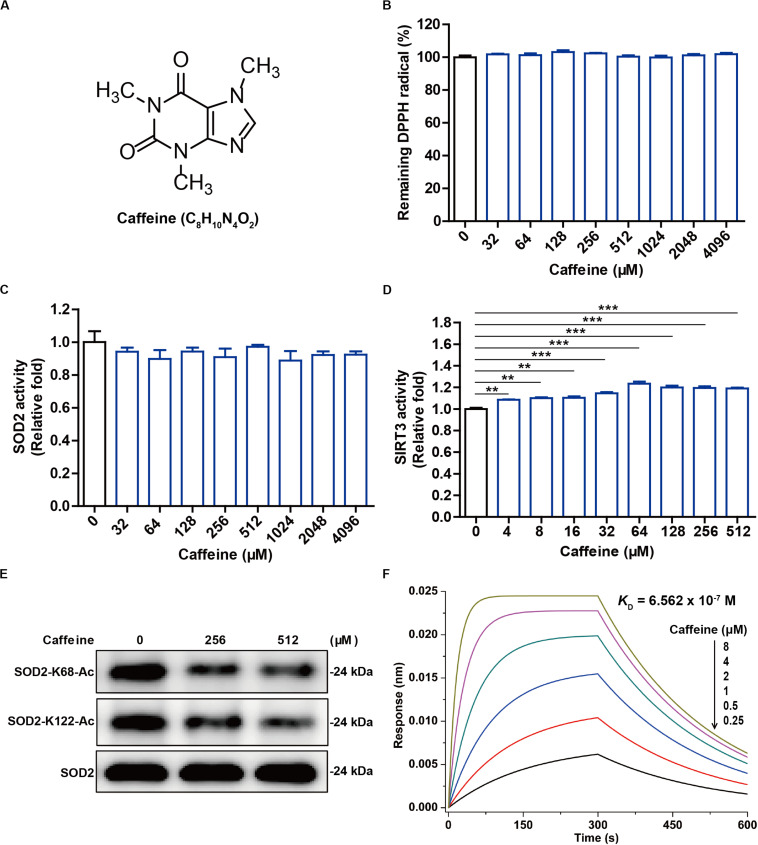
Caffeine has no antioxidant activity *in vitro* but enhances SIRT3 activity. **(A)** The structure of caffeine. **(B)** Caffeine showed no DPPH (free radical) scavenging activity, as evidenced by the absence of any change in absorbance. **(C)** Caffeine does not directly enhance SOD2 activity *in vitro*. **(D)** Caffeine directly enhances SIRT3 activity in an *in vitro* system. **(E)** Caffeine promotes SIRT3-mediated deacetylation of SOD2 *in vitro*. **(F)** Caffeine directly binds to SIRT3 with high affinity (*K*_D_ = 6.562 × 10^– 7^ M). BLI sensorgrams indicating the interactions between gradient concentrations of caffeine and SIRT3 were measured on an Octet Red 96 system, with association and dissociation for 300 s each. Data are expressed as the mean ± SEM of three independent experiments. **P < 0.01, ***P < 0.001 *vs.* the control.

Caffeine is a highly stable alkaloid compound that is not easily oxidized, with methyl groups at the 1, 3, and 7 positions of the purine ring structure (Figure 1A). We evaluated the antioxidant activity of caffeine in DPPH free radical scavenging experiments. The results showed that 32–4,096 μM caffeine did not reduce free radical levels *in vitro* (Figure 1B). It is reported that antioxidant enzyme SOD2 protects against oxidative stress by converting superoxide anion into less toxic hydrogen peroxide (Tao et al., 2010). We further investigated whether caffeine could directly affect SOD2 activity *in vitro*. As expected, adding 32–4,096 μM caffeine to the SOD2 pure enzyme reaction system did not directly alter SOD2 activity (Figure 1C).

It is well known that deacetylation influences the antioxidant activity of SOD2, which can be regulated by the NAD^+^-dependent deacetylase SIRT3 (Tao et al., 2010). Interestingly, we found here that caffeine (4–512 μM) significantly enhanced SIRT3 activity in the pure enzyme reaction system (Figure 1D). Moreover, caffeine (256 and 512 μM) effectively downregulated the protein expression levels of SOD2-K68-Ac and SOD2-K122-Ac in the *in vitro* SIRT3-mediated SOD2 deacetylation assay (Figure 1E). To measure a possible direct interaction between SIRT3 and caffeine, we performed molecular interaction assay using an Octet Red 96 instrument. BLI kinetic measurement showed that SIRT3 had a direct interaction with caffeine and that the binding affinity of caffeine to SIRT3 was 0.6562 μM (Figure 1F). Taken together, these results demonstrate that caffeine does not directly scavenge free radicals and may instead exert an indirect antioxidant effect by acting on SIRT3.

### Caffeine Directly Binds to SIRT3 to Enhance Its Substrate Binding Affinity

**FIGURE 2:**
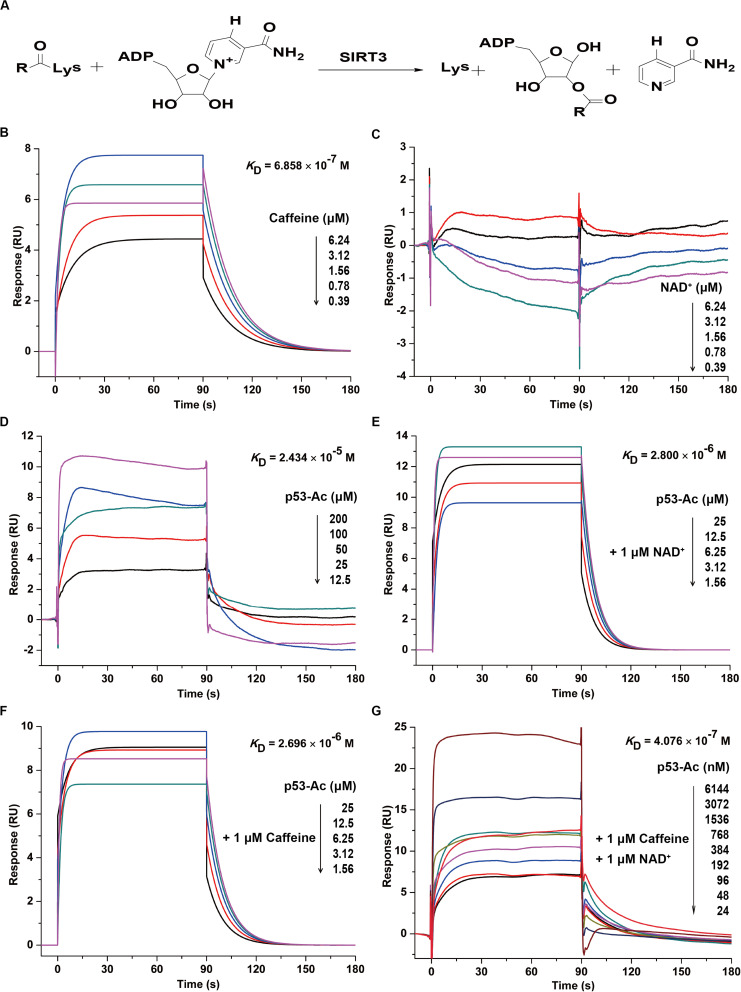
Caffeine directly binds to SIRT3 to enhance its substrate binding affinity. **(A)** Enzymatic reaction involving SIRT3. **(B)** Caffeine binds to SIRT3, with *K*_D_ = 6.858 × 10^– 7^ M. **(C)** NAD^+^ does not directly interact with SIRT3. **(D)** The acetylated substrate p53-Ac binds to SIRT3, with *K*_D_ = 2.434 × 10^– 5^ M. **(E)** NAD^+^ is the coenzyme for the SIRT3 deacetylation reaction, and its presence enhances the binding affinity of p53-Ac to SIRT3, with *K*_D_ = 2.800 × 10^– 6^ M. **(F)** Addition of caffeine enhances the binding affinity between SIRT3 and its substrate, with *K*_D_ = 2.696 × 10^– 6^ M. **(G)** The binding affinity between SIRT3 and its substrate is increased in the presence of caffeine and NAD^+^, with *K*_D_ = 4.076 × 10^– 7^ M. All SPR experiments were carried out using a Biacore S200 instrument (Biacore, GE Healthcare). The data represented here represent one of three independent experiments with similar results.

SIRT3 is an NAD^+^-dependent deacetylase that transfers ADP-ribose from NAD^+^ to a substrate and the acetyl group on the substrate lysine residue to generate nicotinamide (Scher et al., 2007). During catalysis, SIRT3 removes the acetyl group on lysine via NAD^+^ to form 2′-*O*-acyl-ADP-ribose and nicotinamide (Figure 2A). In addition, NAD^+^ binds to and promotes the activity of the enzyme by altering substrate conformation (Jin et al., 2009).

To further confirm whether caffeine directly interacts with SIRT3, we evaluated their real-time interaction using a Biacore S200 SPR system. Consistent with the result of BLI kinetic measurement, caffeine directly bound to SIRT3 with an *K*_D_ of 0.6858 μM (Figure 2B). However, NAD^+^ and SIRT3 did not directly interact (Figure 2C), which is consistent with the result of a previous study (Jin et al., 2009). In addition, SIRT3 directly bound to its substrate acetylated p53 (p53-Ac) with a *K*_D_ of 24.34 μM (Figure 2D). Addition of the coenzyme NAD^+^ (1 μM) increased the binding affinity of p53-Ac to SIRT3, with a *K*_D_ of 2.8 μM (Figure 2E). A similar binding affinity (*K*_D_ = 2.696 μM) between p53-Ac and SIRT3 was observed in the presence of caffeine (1 μM) (Figure 2F). Moreover, when caffeine (1 μM) was added to the complete SIRT3 reaction system, p53-Ac had the strongest affinity (*K*_D_ = 0.4076 μM) for SIRT3 binding (Figure 2G). These data indicated that the binding affinity between SIRT3 and its substrate p53-Ac was 9.03 (without NAD^+^) or 6.87 (with NAD^+^) times higher in the presence of caffeine. Taken together, caffeine can directly bind to SIRT3 with high affinity and enhance its interaction with its substrate p53-Ac.

### Caffeine Reverses UV Irradiation-Induced Oxidative Stress and Apoptosis in HaCaT Cells

**FIGURE 3:**
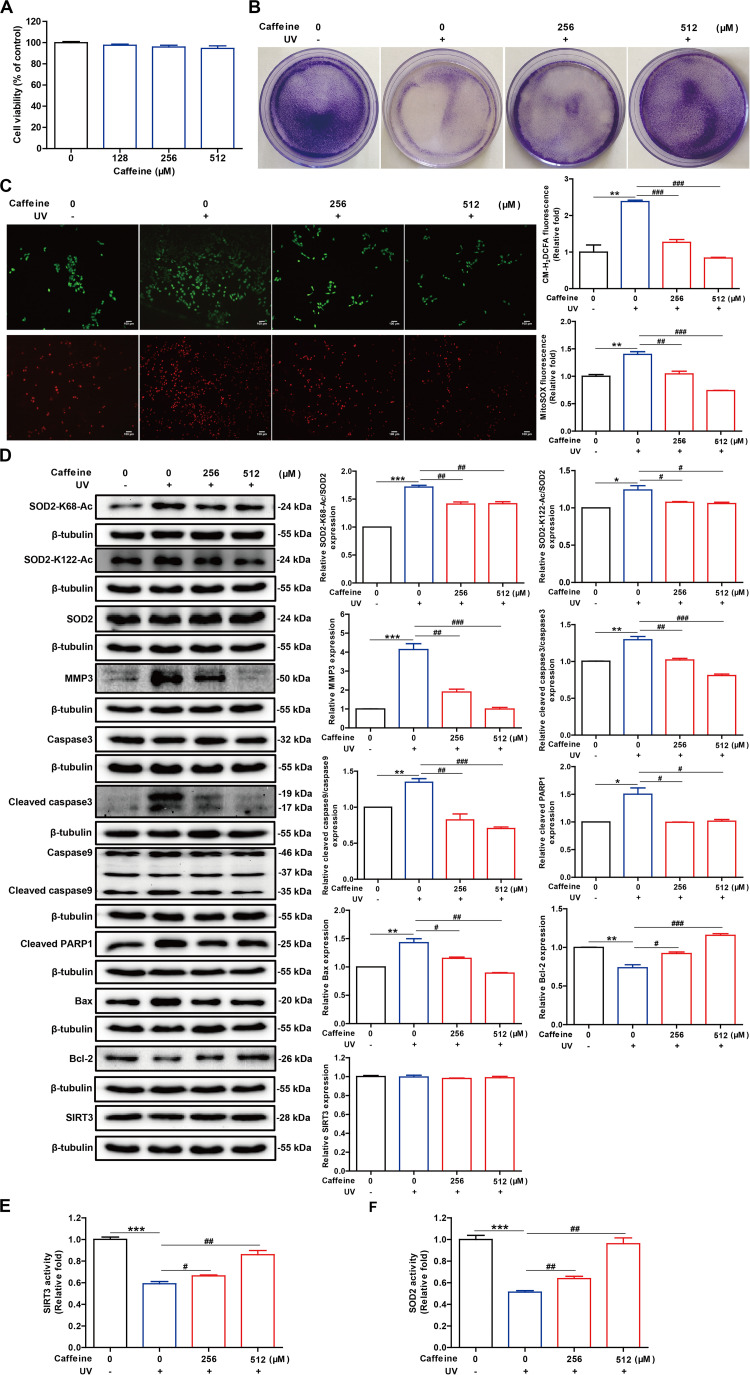
Caffeine pretreatment of HaCaT cells before UV exposure enhances the removal of UV-induced ROS and inhibits apoptosis. **(A)** Caffeine did not affect the viability of HaCaT cells, as determined with the MTT assay. **(B)** Caffeine attenuated the growth inhibitory effect of UV irradiation in HaCaT cells. **(C)** UV-induced intracellular and mitochondrial ROS accumulation in HaCaT cells determined using CM-H_2_DCFDA and MitoSOX, respectively. Images were captured at 200× magnification. **(D)** Western blotting analysis of the HaCaT whole-cell lysates with the indicated antibodies. SIRT3 **(E)** and SOD2 **(F)** activities in the HaCaT whole-cell lysates. Data are expressed as the mean ± SEM of three independent experiments. *P < 0.05, **P < 0.01, ***P < 0.001 *vs.* the control; ^#^*P* < 0.05, ^##^*P* < 0.01, ^###^*P* < 0.001 vs. UV irradiation only.

UV irradiation induces oxidative stress in HaCaT cells, leading to the accumulation of ROS and reducing cellular antioxidant capacity, which has been linked to chronic respiratory organ and skin diseases (Chen et al., 2014; Cavinato and Jansen-Durr, 2017). Since caffeine was reported to protect cells from oxidative stress and can directly enhance SIRT3 activity, we asked if caffeine could exert an indirect antioxidant effect by acting on SIRT3. To confirm the antioxidant effects of caffeine and elucidate the underlying mechanism at the cellular level, we examined changes in oxidative stress and apoptosis markers in UV-irradiated HaCaT cells. We first evaluated the toxicity of different concentrations of caffeine in HaCaT cells with the MTT assay and found that caffeine treatment (128–512 μM) did not affect the viability of HaCaT cells (Figure 3A). Further, crystal violet staining revealed that caffeine at 256 and 512 μM could effectively attenuate the growth inhibitory effect of UV irradiation in HaCaT cells (Figure 3B). Therefore, we selected 256 and 512 μM as the optimal concentrations of caffeine for subsequent *in vitro* experiments.

Given that UV light can induce ROS accumulation, we assessed the levels of intracellular and mitochondrial ROS using the corresponding fluorescent dyes, respectively. The results showed that treatment with caffeine (256 and 512 μM) significantly reduced intracellular and mitochondrial ROS accumulation caused by UV exposure (Figure 3C). Excess ROS in cells stimulates the release of cytochrome C into the cytoplasm, which activates the caspase cascade, leading to apoptosis (Chen et al., 2014; Liu et al., 2017). Flow cytometry analysis showed that the UV irradiation-induced apoptosis was significantly inhibited following caffeine treatment (Supplementary Figure 1). As expected, Caffeine treatment significantly inhibited UV irradiation-induced expression levels of MMP3, cleaved caspase 3, cleaved caspase 9, cleaved PARP1, and Bax in HaCaT cells. Additionally, caffeine treatment significantly increased the expression level of the antiapoptotic protein Bcl-2 in UV-irradiated HaCaT cells (Figure 3D). These results revealed that UV radiation-induced apoptosis was significantly reduced in the presence of caffeine.

Previous molecular interaction experiments have shown that caffeine directly binds to SIRT3 with high affinity and enhances its interaction with substrates, thereby enhancing SIRT3 activity *in vitro*. At the same time, SOD2 is the deacetylated substrate of SIRT3 whose acetylation level is closely related to its antioxidant activity (Tao et al., 2010; Zhu et al., 2019). As such, we further assessed the activities of SOD2 and SIRT3 in cell lysates and found that treatment with caffeine significantly enhanced the activities of both enzymes (Figures 3E,F). Of note, caffeine treatment had no effect on the protein expression levels of SOD2 and SIRT3 in UV-irradiated HaCaT cells (Figure 3D). More importantly, western blotting experiments showed that caffeine treatment significantly decreased UV irradiation-induced SOD2 acetylation (Figure 3D). Taken together, these results indicate that caffeine enhances SOD2 activity by modulating the acetylation of SOD2 by SIRT3, thereby suppressing oxidative stress and apoptosis caused by UV radiation.

### SIRT3 Inhibitor 3-TYP Abrogates the Protective Effect of Caffeine in UV-Irradiated HaCaT Cells

**FIGURE 4:**
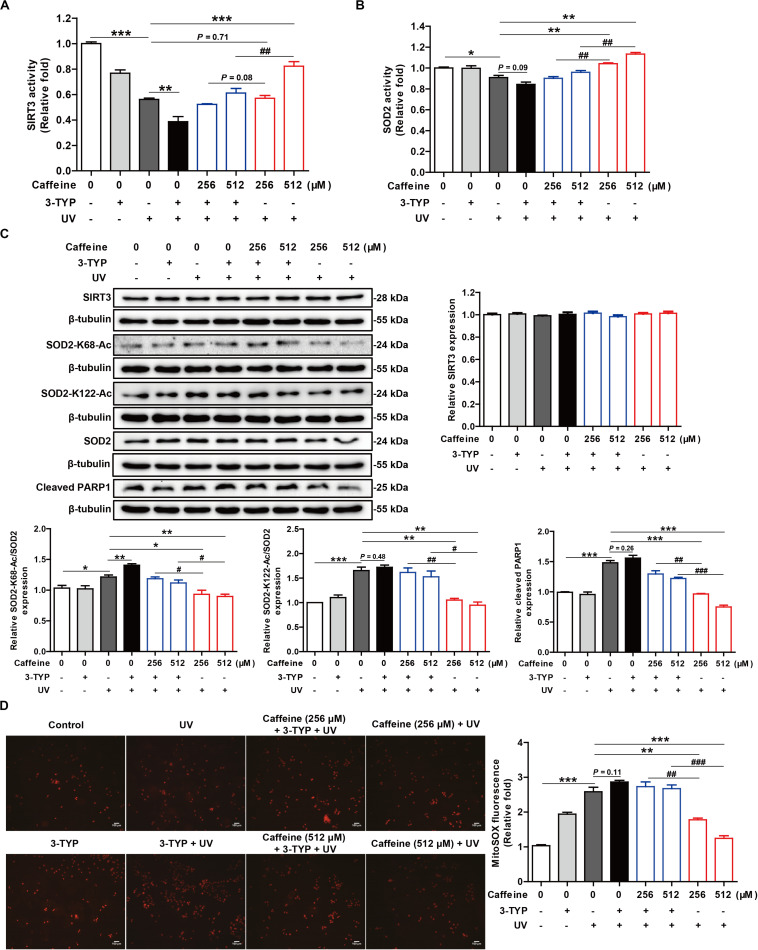
SIRT3 inhibitor 3-TYP abrogates the protective effect of caffeine in UV-irradiated HaCaT cells. Detection of SIRT3 **(A)** and SOD2 **(B)** activities. **(C)** Western blotting analysis of the HaCaT whole-cell lysates with the indicated antibodies. **(D)** Detection of mitochondrial ROS. Images were captured at 200× magnification. Data are expressed as the mean ± SEM of three independent experiments. *P < 0.05, **P < 0.01, ***P < 0.001 vs. UV group; ^#^*P* < 0.05, ^##^*P* < 0.01, ^###^*P* < 0.001 vs. “Caffeine (1.29 mM) + UV” group or “Caffeine (5.15 mM) + UV” group.

To confirm that SOD2 acetylation is regulated by SIRT3 and that caffeine exerts its antioxidant effects through acting on SIRT3, HaCaT cells were treated with 3-TYP, a SIRT3 inhibitor, in the presence of caffeine. After UV irradiation, the activities of SIRT3 and SOD2 were markedly decreased in cells co-treated with caffeine and 3-TYP compared to caffeine alone (Figures 4A,B), suggesting that caffeine targets SIRT3 to enhance SOD2 activity. Western blot analysis further revealed that the protein expression levels of SOD2-K68-Ac, SOD2-K122-Ac, and cleaved PARP1 after addition of caffeine and 3-TYP were higher than in UV-irradiated cells treated with caffeine only (Figure 4C). Notably, there were no differences observed in the protein expression levels of SIRT3 and SOD2 in any of the groups (Figure 4C). Moreover, the SIRT3 inhibitor 3-TYP significantly increased mitochondrial ROS accumulation in UV-irradiated cells treated with caffeine (Figure 4D). Collectively, these results demonstrate that the protective effects of caffeine against UV radiation-induced oxidative stress are dependent on SIRT3.

### Caffeine Protects Skin From UV Damage *in vivo* by Acting on SIRT3

**FIGURE 5:**
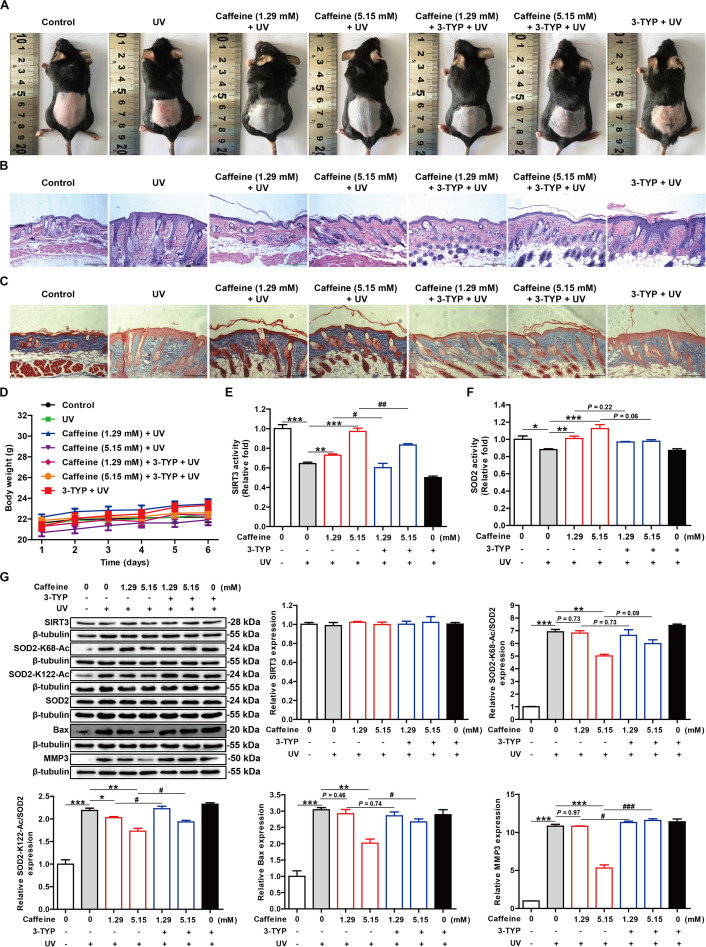
Caffeine protects mouse skin from UV damage by acting on SIRT3. **(A)** Appearance of the dorsal skin from mice. **(B,C)** Histological analysis of skin sections by H&E and Masson’s trichrome staining. Images were captured at 100× magnification. **(D)** Body weight of mice. SIRT3 **(E)** and SOD2 **(F)** activities in mouse skin tissues. **(G)** Western blotting analysis of the mouse skin tissues with the indicated antibodies. Data are expressed as the mean ± SEM of six mice per group. *P < 0.05, **P < 0.01, ***P < 0.001 vs. UV group; ^#^*P* < 0.05, ^##^*P* < 0.01, ^###^*P* < 0.001 vs. “Caffeine (1.29 mM) + UV” group or “Caffeine (5.15 mM) + UV” group.

To further verify the antioxidant effects of caffeine and its molecular mechanism *in vivo*, we used a mouse model of skin photoaging. The exposed dorsal skin of mice was treated with caffeine 1 h before UV irradiation, and photographs of the skin were acquired at the end of animal experiment. The skin of mice treated with caffeine had fewer wrinkles and dander than that of UV-irradiated mice, and showed less redness (Figure 5A). H&E staining of skin sections revealed an increased thickness of the stratum corneum and damage to the dermis caused by acute UV irradiation, and this phenomenon could be ameliorated by caffeine treatment (Figure 5B). Additionally, Masson’s trichrome staining showed that UV irradiation caused the degradation of collagen in skin, which was mitigated by caffeine treatment (Figure 5C). As expected, the protective effect of caffeine on UV-irradiated mouse skin was abrogated by application of the SIRT3 inhibitor 3-TYP (Figures 5A–C). In addition, there was no difference observed in body weight in any of the groups (Figure 5D).

To further confirm the molecular basis of the above observations, we evaluated the activities of SOD2 and SIRT3 extracted from skin tissue. Consistent with the *in vitro* results, treatment with caffeine significantly increased SIRT3 and SOD2 activities in UV-irradiated mouse skin, which could be partly reversed by the addition of 3-TYP (Figures 5E,F). TUNEL staining showed that caffeine treatment effectively inhibited apoptosis in UV-irradiated mouse skin and 3-TYP treatment could weaken this effect (Supplementary Figure 2). Western blot analysis revealed an increase in SOD2-K68-Ac and SOD2-K122-Ac levels along with upregulation of Bax and MMP3 proteins induced by UV irradiation, which could be inhibited by caffeine treatment (Figure 5G). As expected, 3-TYP treatment increased the protein expression levels of SOD2-K68-Ac, SOD2-K122-Ac, Bax, and MMP3 in UV-irradiated mouse skin treated with caffeine (Figure 5G). Similarly, there were no differences observed in the protein expression levels of SIRT3 and SOD2 in any of the groups (Figure 5G). Taken together, the above results strongly demonstrate that caffeine exerts protective effects against UV-induced skin damage through acting on SIRT3.

## Discussion

A major cause of cellular senescence and numerous aging-related diseases is thought to be cumulative oxidative stress, resulting from the production of ROS (Qiu et al., 2010). Free radicals are metabolic intermediates produced during normal physiological reactions, but an excess of ROS or a diminished antioxidant capacity can lead to oxidative stress and related diseases such as aging and inflammation (Lu et al., 2017; Li et al., 2018). Skin provides a protective envelope between an organism and the environment that is crucial for homeostasis (Baek et al., 2017). Studies indicate that oxidative stress plays a key role in the development of skin aging, because it can seriously impair the mitochondrial function, which in turn promotes excessive generation of ROS to accelerate the senescence process (Harman, 1992; Chen et al., 2014; Cavinato and Jansen-Durr, 2017). It is well known that the skin barrier integrity can be impaired by solar UV rays and ROS, and excess exposure to UV radiation contributes to acute skin damage including epidermal injury (Kim, 2016; Reelfs et al., 2016; Das et al., 2019).

Antioxidant compounds as vitamins C and E have potent reducing activity due to their characteristic chemical structures, which can directly scavenge free radicals (Amir Aslani and Ghobadi, 2016). Caffeine, as an very important natural secondary metabolite, occurs in some plant leaves, seeds, fruits, and barks (Lelo et al., 1986; Verster and Koenig, 2018), where it can serve as an insect repellent, herbicide, and even attractant for pollination (Wikoff et al., 2017). More importantly, this botanically sourced compound has attracted widespread attentions from humans due to its broad-spectrum pharmacological activities across a wide range of doses (Cano-Marquina et al., 2013; Verster and Koenig, 2018; Acidri et al., 2020). Caffeine is generally recognized as safe by the Food and Drug Administration and approximately 87% of the world’s population consumes an average of 193 mg of caffeine every day (Huang et al., 2014). Intriguingly, caffeine has a stable chemical structure that is not easily reduced. Thus, it should not possess the antioxidant activities, which is consistent with the result of the DPPH assay in this study. Nonetheless, caffeine has antioxidant properties that protect skin cells from oxidative stress-induced senescence (Li et al., 2018). This paradox described above prompted us to speculate that caffeine may indirectly exert antioxidant function mainly by regulating an intrinsic molecular mechanism.

It has been reported that antioxidant and redox signaling events are rigidly regulated by some critical molecules that modulate antioxidants, ROS, and/or oxidative stress within the mitochondria (Chen et al., 2014; Singh et al., 2018). Imbalances in these molecules can directly disturb mitochondrial functions to become pathogenic (Singh et al., 2018; Ping et al., 2020). SIRTs serve as important regulators of redox homeostasis in cells (Singh et al., 2018). Studies show that SIRT1, SIRT3, and SIRT5 can protect the cell from ROS, and SIRT2, SIRT6, and SIRT7 can regulate key oxidative stress genes and mechanisms (Herskovits and Guarente, 2013; Jing and Lin, 2015; Singh et al., 2018). Interestingly, SIRT4 has been shown to induce ROS production but also has antioxidative roles as well (Singh et al., 2018). Among these SIRTs, SIRT3 is the most thoroughly studied NAD^+^-dependent mitochondrial deacetylase, and plays vital roles in maintaining redox homeostasis (Yang et al., 2016; Hershberger et al., 2017; Liu et al., 2017). In the present study, we investigated whether the antioxidant effects of caffeine involve SIRT3 using *in vitro* and *in vivo* models. Intriguingly, we showed here that caffeine could directly enhance the catalytic activity of SIRT3 *in vitro* and *in vivo*. As for the detailed mechanisms, SPR studies demonstrated that caffeine could directly bind to SIRT3 with high affinity (*K*_D_ = 0.6858 μM), and significantly increase the binding affinity between SIRT3 and its substrate p53-Ac in the absence or presence of NAD^+^. In addition, the binding study by SPR suggests that the acetylated peptide is the first substrate to bind to SIRT3, before NAD^+^, which is consistent with the result of a previous ITC study (Jin et al., 2009).

Reactive oxygen species are produced as a natural byproduct of cellular respiration. Antioxidant enzymes, especially SODs, can scavenge ROS and maintain a reducing environment in the skin cell. An imbalance between the generation of ROS and the skin cell’s ability to readily detoxify ROS can disturb the redox homeostasis and result in oxidative stress (Papaconstantinou, 2019; Ungvari et al., 2019; Gu et al., 2020). Notably, in the UV irradiated skin cells, the activities of SIRT3, a mitochondrial deacetylase, and SOD2, a major mitochondrial antioxidant enzyme, were significantly inhibited (Tao et al., 2010; Lu et al., 2017). More importantly, it has been verified that SIRT3 can deacetylate SOD2 to augment its antioxidative capacity (Qiu et al., 2010; Liu et al., 2017). In this regard, restoring SIRT3 activity can protect skin from oxidative stress- or UV-induced damage. Interestingly, we found that caffeine could effectively protect skin from UV-induced damage *in vitro* and *in vivo* via enhancing the activities of SIRT3 and SOD2, and the antioxidant effects of caffeine could be abolished by treatment with the SIRT3 inhibitor 3-TYP.

Mitochondria play a pivotal role in apoptosis through releasing cytochrome c and other proapoptotic proteins (Sena and Chandel, 2012; Baek et al., 2017). In addition, aberrant mitochondrial ROS levels are thought to be closely associated with activation and propagation of apoptosis (Sena and Chandel, 2012; Shadel and Horvath, 2015). Mitochondria are the major source of ROS in UV-irradiated skin cells. In the present study, we found that caffeine could significantly scavenge mitochondrial ROS and inhibit the expression levels of some proapoptotic proteins in UV-irradiated HaCaT keratinocytes. Additionally, caffeine could reverse the decreased SIRT3 and SOD2 activities and the increased SOD2 acetylation levels in UV-irradiated HaCaT cells. Consistently, *in vivo* studies further showed that smear administration of caffeine could effectively protect skin from UV-induced damage through increasing SIRT3 and SOD2 activities. Thus, these results demonstrate that caffeine directly stimulates the deacetylase activity of SIRT3 and thereby reduces the acetylation level of SOD2 to restore its antioxidant activity, which ultimately prevents cellular damage caused by UV radiation-induced ROS production.

Notably, the primary cellular target of caffeine is the adenosine receptor A2AR, and a recent study revealed that inhibition of A2AR by caffeine activated the SIRT3/AMPK-regulated autophagy/mitophagy pathway to relieve oxidative stress (Li et al., 2018). However, the present study demonstrates for the first time that the mitochondrial deacetylase SIRT3 is a new binding target protein of caffeine. Our data indicate that caffeine acts as a small-molecule activator to promote the SIRT3-enzymatic reaction, thereby preventing acute skin damage under pathophysiological conditions such as excess exposure to UV radiation. To our knowledge, extremely few natural small-molecule compounds are known to bind strongly to SIRT3 as an activator. Although we obtained the above results, the binding site of caffeine to SIRT3 remains unclear and warrants further investigation. Additionally, given that caffeine can cross the blood-brain barrier and SIRT3 plays a vital role in manipulating oxidative stress in various cells and tissues (Yeh et al., 2014; Chan et al., 2016; Khan et al., 2019), we speculate that caffeine can target SIRT3 to protect against other oxidative stress-induced diseases, such as liver damage, diabetes, and neurological diseases. However, this hypothesis requires further verification in future studies.

## Conclusion

In summary, the findings of this study demonstrate that caffeine targets SIRT3 to enhance SOD2 activity and protect skin cells from UV irradiation-induced oxidative stress. Thus, caffeine, as a small-molecule SIRT3 activator, could be a potential agent to protect human skin against UV radiation.

## Data Availability Statement

All datasets generated for this study are included in the article/Supplementary Material.

## Ethics Statement

The animal study was reviewed and approved by the Animal Care and Use Committee of Yunnan Agricultural University (Approval Number: YNAU2019LLWYH003).

## Author Contributions

JS, XW, and HX conceived and designed the experiments. CG, HX, DZ, ZG, YH, and SW performed the experiments. HX, CG, and DZ analyzed the data. JS and XW contributed reagents, materials, and analysis tools. HX and CG wrote the manuscript. All authors read and approved the final manuscript.

## Conflict of Interest

The authors declare that the research was conducted in the absence of any commercial or financial relationships that could be construed as a potential conflict of interest.

## Abbreviations

3-TYP: 3-(1H-1,2,3-triazol-4-yl) pyridine
A2AR: adenosine A2a receptor
AMPK: adenosine monophosphate protein kinase
Bax: Bcl-2-associated X protein
Bcl-2: B cell lymphoma 2
BLI: biolayer interferometry
CM-H_2_DCFDA: 5-(6)-chloromethyl-2 ′, 7 ′ -dichlorodihydrofluorescein diacetate acetyl ester
DMEM: Dulbecco’s Modified Eagle’s Medium
DMSO: dimethyl sulfoxide
DPPH: 2,2-diphenyl-1-picrylhydrazyl
H&E: hematoxylin and eosin
*K*_D_: equilibrium dissociation constant
MMPs: matrix metalloproteinases
MTT: 3-(4,5-dimethylthiazol-2-yl)-2,5-diphenyltetrazolium
NAD^+^: nicotinamide adenine dinucleotide
PI: propidium iodide
ROS: reactive oxygen species
SEM: standard error of the mean
Sir2: silent information regulator 2
SIRT3: sirtuin 3
SOD2: superoxide dismutase 2
SPR: surface plasmon resonance
UV: ultraviolet.
