# Supplementary material for: Caffeine Targets SIRT3 to Enhance SOD2 Activity in Mitochondria
Source: Front Cell Dev Biol. 2020 Sep 1;8:822. doi: 10.3389/fcell.2020.00822 (PMC7493682; doi:10.3389/fcell.2020.00822)
Supplement: Supplementary file 1 [file Image_1.pdf]

Supplementary Material

Caffeine Targets SIRT3 to Enhance SOD2 Activity in Mitochondria

Huanhuan Xu[1,2†], Chunxia Gan[1,3†], Ziqi Gao[1,3], Yewei Huang[1,2], Simin Wu[1,3], Dongying Zhang[1,2*], Xuanjun Wang[1,2,4]* and Jun Sheng[1,4]*

***Correspondence to:** Key Laboratory of Pu-er Tea Science, Ministry of Education, Yunnan Agricultural University, No. 452, Fengyuan Road, Panlong District, Kunming 650201, China. E-mail addresses: 757664982@qq.com (Dongying Zhang); jwang@ynau.edu.cn (Xuanjun Wang); shengj@ynau.edu.cn (Jun Sheng).

[†]These authors contributed equally to this work.

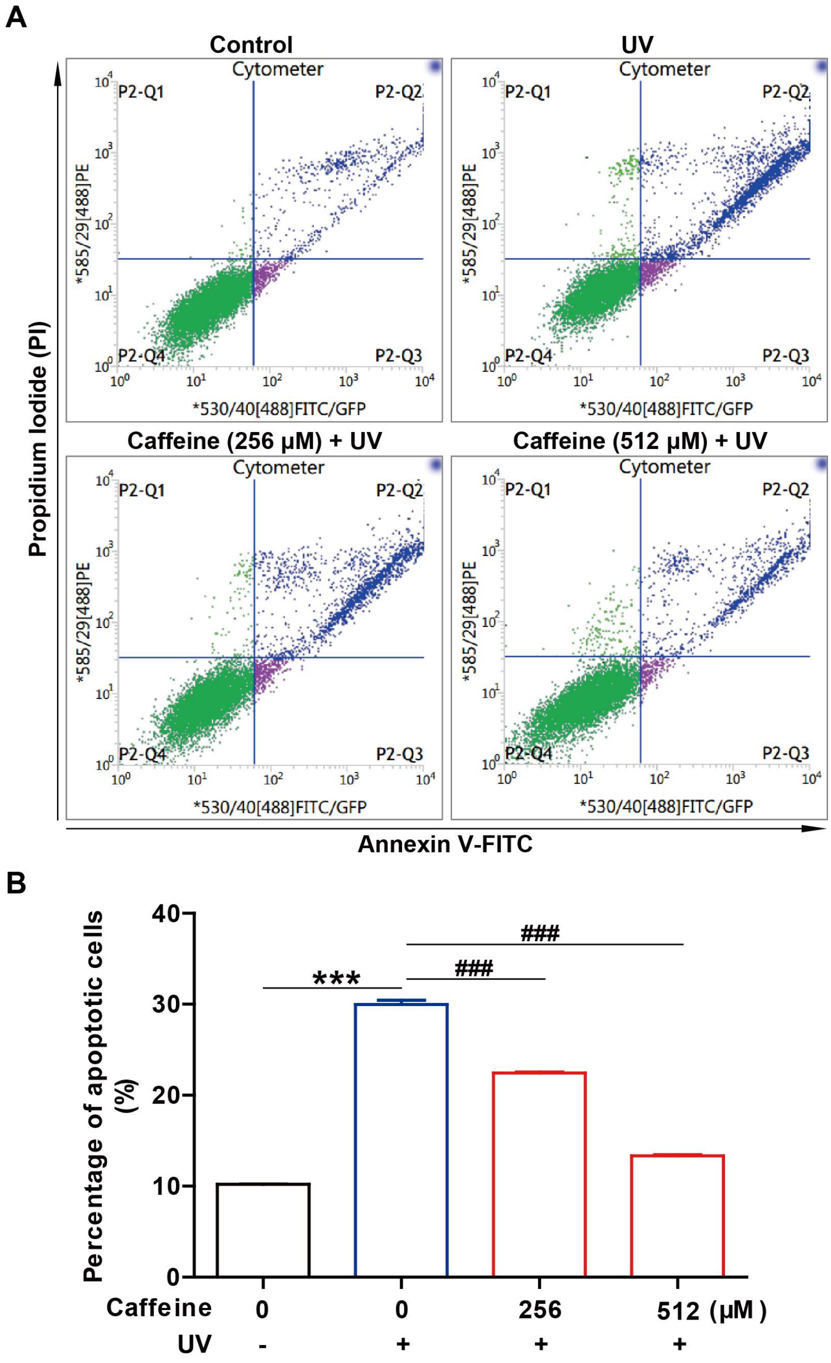

Supplementary Figure 1. Caffeine inhibits UV irradiation-induced apoptosis in HaCaT cells. **(A)** Flow cytometry was used to detect cell apoptosis in UV-irradiated HaCaT cells treated with various concentrations of caffeine. **(B)** The ratio of apoptotic cells in each group are expressed as percentages. Data are expressed as the mean ± SEM of three independent experiments. $^{***}P < 0.001$ *vs.* the control; $^{###}P < 0.001$ *vs.* UV irradiation only.

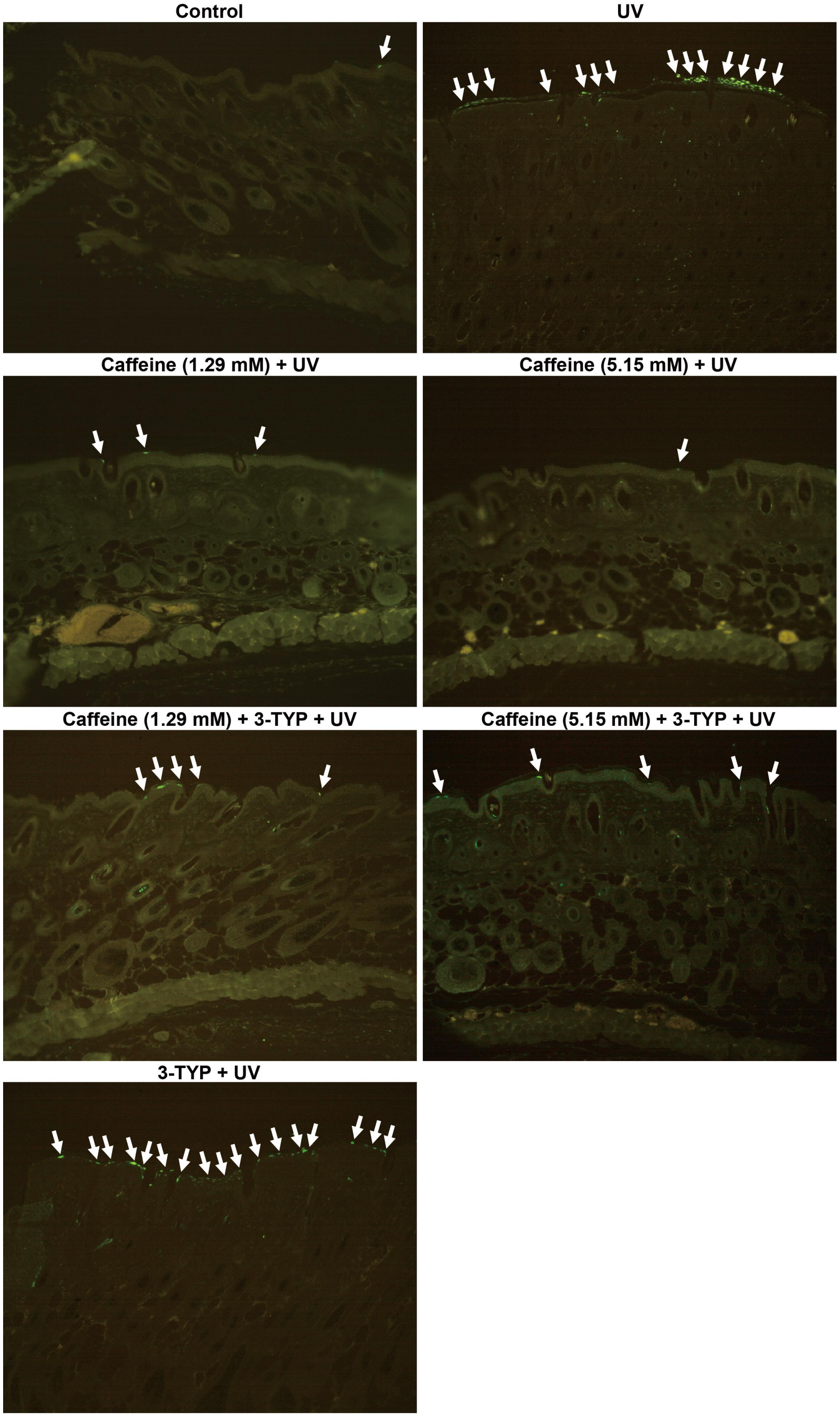

Supplementary Figure 2. Caffeine effectively inhibits apoptosis in UV-irradiated mouse skin. Apoptosis in mouse skin sections was examined by TUNEL staining and images were captured at ×100 magnification. The green fluorescence represents apoptotic cells.
